# Challenges in Acceptance and Compliance in Digital Health Assessments During Pregnancy: Prospective Cohort Study

**DOI:** 10.2196/17377

**Published:** 2020-10-14

**Authors:** Katharina Brusniak, Hannah Maria Arndt, Manuel Feisst, Kathrin Haßdenteufel, Lina Maria Matthies, Thomas Maximilian Deutsch, Hannes Hudalla, Harald Abele, Markus Wallwiener, Stephanie Wallwiener

**Affiliations:** 1 Department of Gynecology and Obstetrics University Hospital Heidelberg Heidelberg Germany; 2 Institute of Medical Biometry and Informatics Heidelberg University Heidelberg Germany; 3 Department of Neonatology University Hospital Heidelberg Heidelberg Germany; 4 Department of Women's Health University Hospital Tübingen Tübingen Germany; 5 Section of Midwifery Science Institute for Health Sciences University Hospital Tübingen Tübingen Germany

**Keywords:** eHealth, compliance, pregnancy, digital assessments

## Abstract

**Background:**

Pregnant women are increasingly using mobile apps to access health information during the antenatal period. Therefore, digital health solutions can potentially be used as monitoring instruments during pregnancy. However, a main factor of success is high user engagement.

**Objective:**

The aim of this study was to analyze engagement and factors influencing compliance in a longitudinal study targeting pregnant women using a digital health app with self-tracking.

**Methods:**

Digitally collected data concerning demographics, medical history, technical aspects, and mental health from 585 pregnant women were analyzed. Patients filling out ≥80% of items at every study visit were considered to be highly compliant. Factors associated with high compliance were identified using logistic regression. The effect of a change in mental and physical well-being on compliance was assessed using a one-sample *t* test.

**Results:**

Only 25% of patients could be considered compliant. Overall, 63% left at least one visit blank. Influential variables for higher engagement included higher education, higher income, private health insurance, nonsmoking, and German origin. There was no relationship between a change in the number of physical complaints or depressive symptoms and study dropout.

**Conclusions:**

Maintaining high engagement with digital monitoring devices over a long time remains challenging. As cultural and socioeconomic background factors had the strongest influence, more effort needs to be directed toward understanding the needs of patients from different demographic backgrounds to ensure high-quality care for all patients. More studies need to report on compliance to disclose potential demographic bias.

## Introduction

Pregnant women make up a significant proportion of the world’s population. With an average age of 30 years, pregnant women represent a generation of patients eager to experience new technologies and extend medical care in the digital sector [1-3]. The use of pregnancy apps among expectant mothers is high; such apps can therefore either be used as educational devices or as monitoring instruments during pregnancy, based on regular assessments of patient-reported outcomes (PROs), as well as psychological and physical symptoms [4,5].

A PRO is defined as “a measurement based on a report that comes directly from the patient (i.e., study subject) about the status of a patient’s health condition without amendment or interpretation of the patient’s response by a clinician or anyone else” [6]. Many factors such as body mass index (BMI), physical symptoms, or even depressive symptoms can be assessed using PROs or validated questionnaires. Self-reporting of information concerning delivery or prepregnancy weight were reported to be of high accuracy [7,8].

Furthermore, digital monitoring devices could offer a benefit to women at risk for preterm birth due to a reduction of adverse fetal outcomes and costs [9]. Electronic health solutions offer not only the possibility of self-tracking, but also health education. Digital self-tracking of uterine activity has already been shown to prolong pregnancy and improve outcomes for the baby. Online education on smoking and nutrition can also have significant impact on patients’ behavior [9,10]. Thus, digital solutions during pregnancy have the potential to improve efficiency and quality of care [11]. With the growing demand of easily accessible educational health information, tailored interventions, and more personalization, digital health apps offer unique opportunities and may be beneficial to treatment compliance [1,12]. In addition to that, there have been reports of higher use of pregnancy apps among women with depression, a history of chronic illness, or other risk factors for adverse outcomes, including smoking [3,13,14].

Thus, electronic health (eHealth) apps seem to be the perfect fit for obstetrics, with great potential for modifying the structure of perinatal care [15]. Several online information platforms or pregnancy apps for expectant mothers and their partners exist at present, providing information as well as online coaching, with good rates of compliance and success in lifestyle interventions [16]. However, up to now, there have been no digital prevention programs routinely integrated into antenatal care.

To achieve successful integration into routine care, high patient engagement, also referred to as compliance, remains a key factor. Compliance is defined as the “the consistency and accuracy with which a patient follows the regimen prescribed by a physician or other health care professional” [17]. In any digital health program, compliance is a key challenge and an essential factor for a successful outcome, as low compliance can threaten the validity of a study [18]. A substantial number of patients stop using apps before the completion of a program [19]. Several studies from different fields reported that compliance declined through the course of their study [20,21]. However, the definition of compliance is often not clarified or can vary greatly among different studies according to the study format. In addition to that, not all studies report on compliance, but rather dropout rates of presumably official study dropouts.

In addition to that, people from lower socioeconomic backgrounds or of minority ethnicities have been reported to have lower compliance rates [18,20-22]. There have also been reports that patients with poorer lifestyle and health profiles are less compliant [18].

To reach those marginal groups of patients at a presumed risk of adverse pregnancy outcomes, some mobile apps have already received broad acceptance as a way of low-threshold, interactive care [23]. In contrast, apps may not effectively engage “hard-to-reach” groups, such as women of low income and those with lower levels of education [24]. Nonetheless, incentives or reminders can be implemented to effectively increase compliance [25,26].

Therefore, this study aimed to examine compliance and its influencing factors among pregnant women in a prospective cohort study. This analysis focused on characterizing women with high engagement and investigating potential influencing factors to understand patients’ engagement in digital apps as a key factor for successfully integrating eHealth in routine care settings.

## Methods

### Participants and Study Design

**Figure 1:**
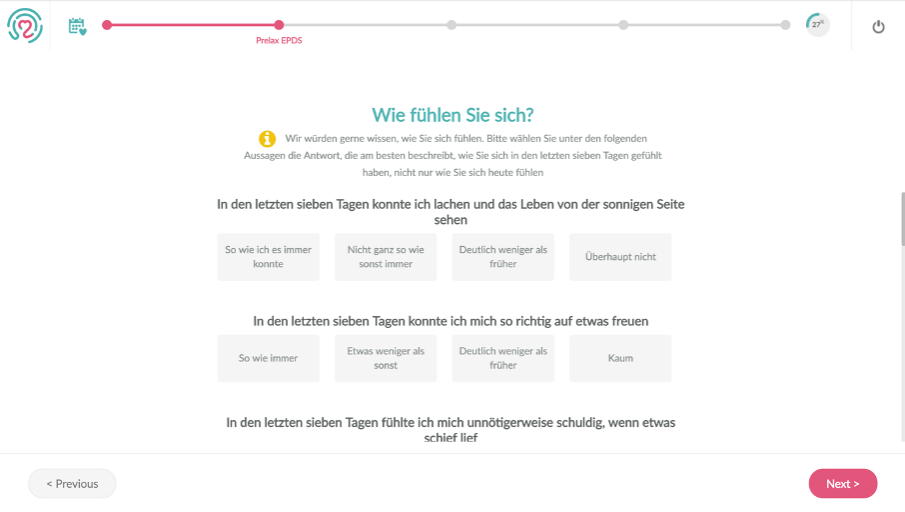
Screenshot of the PiiA platform. PiiA: Patient-informiert-interaktiv-Arzt (patient-informs-interactive-physician).

This exploratory bicentric trial was conducted prospectively between October 2016 and September 2018 at two German university hospitals (University Hospitals of Heidelberg and Tuebingen). It was designed as a longitudinal, bicentric trial that included several questionnaires, which were delivered via an online platform called PiiA (Patient-informiert- interaktiv-Arzt; Figure 1) [27]. Originally, the study design included the randomization of the participants into two groups, with the intervention group having access to an educational pregnancy guidebook in addition to the survey. However, due to the low usage of the app, we refrained from a direct group comparison and treated both groups as one study cohort.

The online study visits and the corresponding individual questionnaires were scheduled monthly until delivery, followed by 3 postnatal visits finishing at 6 months postpartum. In addition to validated questionnaires, various pregnancy-related symptoms and complications were surveyed as part of a self-tracking app. The questionnaires included in this analysis are shown in Table 1.

**Table 1:** Structure of the prenatal section.

Characteristics and questionnaires	Baseline	Visit 1	Visit 2	Visit 3	Visit 4	Visit 5
Gestational age (weeks)	19	20	24	28	32	36
Socioeconomic questionnaire	✓					
Previous medical history	✓					
Physical symptoms		✓	✓	✓	✓	✓
Digital evaluation	✓			✓		
Edinburgh Postnatal Depression Scale		✓	✓	✓	✓	✓
State-Trait Anxiety Inventory			✓		✓	

Participants were enrolled during outpatient prenatal checkups or, in individual cases, during a hospital stay, and were eligible for participation if they were older than 18 years, they had a sufficient level of German language proficiency, they had internet access, and gestational age was between 19 and 27 weeks. Exclusion criteria were inability to understand the content of the study, as well as multiples or chromosomal aberrations and genetic conditions or fetal abnormalities of different or unknown origin.

All study-related contacts took place in addition to standard antenatal care. The study was entirely conducted in the German language. Ethics approval was granted by the Ethical Committee of the University of Heidelberg (S-158/2016) and the University of Tuebingen (062/2017BO2).

### Initial Support and Online Tutorial

After giving informed consent, all patients received a tablet and were introduced to the platform by trained staff who were available for further questions. The baseline visit was filled out onsite. The platform also provided a tutorial that could be accessed by the participant at any time. After the first visit, patients continued the study at home using their own preferred devices. At first login, patients were asked to provide their current gestational week as a trigger for all following visits and reminders, ensuring the exact start of new visits on their respective gestational week.

### Measurements

#### Demographics, Anamnesis, and Physical Complaints

The socioeconomic questionnaire (SEQ) is a self-designed questionnaire that encompasses several items related to demographic characteristics such as age, origin, and education. For retrieving the participants’ medical history, we used a self-designed questionnaire that included a series of obstetric questions on parity, preexisting conditions, and medical conditions in previous pregnancies. The questionnaire contains a selection of common complications and medical diagnoses. Questions concerning technical abilities and preferences were also administered. Furthermore, a questionnaire with a selection of pregnancy-related PRO symptoms was available at every visit as part of a self-tracking app.

#### Edinburgh Postnatal Depression Scale

Depressive symptoms were assessed using the Edinburgh Postnatal Depression Scale (EPDS) at every visit through an overall summary score. The EPDS was originally developed by Cox et al [28] and translated into German by Bergant et al [29]. The EPDS offers high sensitivity and specificity in predicting depressive disorders [29] and has proven to accomplish this in the prenatal and postnatal period [29,30].

#### State-Trait Anxiety Inventory

The State-Trait Anxiety Inventory (STAI) is a two-part questionnaire, each consisting of a summary score of 20 items, to evaluate anxiety as a temporary condition (state, STAI-S) and as a personality characteristic (trait, STAI-T) [31]. We used the German version of the questionnaire [31]. The STAI has proven to be a valid instrument for assessing anxiety in pregnant women [32]. We implemented the STAI at two visits.

### Patient Engagement and Compliance Monitoring

A range of engagement strategies were employed, designed to encourage participants to continue the study. Participants were reminded via email 2 days prior to as well as 3 and 5 days after the scheduled visit date. The rate of completion for each assessment was calculated, measured by the amount of completed questions divided by all available questions at a specific visit. This rate was assessed weekly for each patient that had been reminded in the previous week. If the completion rate was ≤80%, email reminders were sent and escalated to follow-up phone calls if patients did not respond. Patients who officially dropped out of the study were considered as noncompliant patients and were included in the analysis.

### Statistical Analysis

We used the programming language R (Version 3.5.1; R Foundation for Statistical Computing) for all of our analyses [33]. Socioeconomic and obstetric data as well as completion rates were analyzed descriptively by calculating mean scores and standard deviations as well as absolute and relative frequencies. We regarded a completion rate of ≥80% per visit throughout the study as compliant.

First, we performed univariate logistic regression to examine the effect of unique items on compliance. Multivariate logistic regression was then applied to identify influencing factors on compliance ≥80%, using all previously significant variables.

Second, we evaluated whether changes in mental or physical well-being prompted an abrupt discontinuance of study participation. Thereupon, we performed a one-sample *t* test on each change in EPDS score and the number of physical complaints between the last two visits before dropout (decline of completion rate ≥80% followed by no further activity). This analysis included both official dropouts as well as patients concluding the study without further contact or notice. *P* values ≤.05 were considered significant. Since this is an exploratory study, no adjusting for multiplicity was performed and *P* values have to be interpreted in a descriptive sense.

## Results

### Sample Characteristics

This analysis is based on 585 participants pregnant with singletons. In total, 41 patients (7.0%) actively decided to terminate the study before completion, mostly due to personal issues concerning time management, difficulties related to pregnancy, or family reasons. Overall, 319 (54.5%) participants stopped processing the online visits without officially withdrawing their participation in the study and did not respond to further contact attempts by the study staff. The sample characteristics are presented in Table 2.

**Table 2:** Demographic characteristics of the study population.

Variable			Frequency
Age (years), median (Q1-Q3)			33 (29-36)
**BMI, n (%)**			
	<25	333 (60.5)	
	25-30	106 (19.3)	
	>30	111 (20.2)	
**Smoker, n (%)**			
	Current or former	207 (37.4)	
	Never	347 (62.6)	
**Origin, n (%)**			
	German	470 (83.9)	
	Other	90 (16.1)	
**Education, n (%)**			
	University entrance qualification or higher	239 (42.7)	
	Lower than university entrance qualification	321 (57.3)	
**Net monthly income per household (€), n (%)**			
	<1000 (<US $1184)	75 (13.8)	
	1000-2000 (US $1184-$2367)	176 (32.4)	
	2000-3000 (US $2367-$3551)	113 (20.8)	
	>3000 (>US $3551)	180 (33.1)	
**Current employment, n (%)**			
	Yes	469 (83.9)	
	No	90 (16.1)	
**Health insurance, n (%)**			
	Public	435 (77.7)	
	Private	125 (22.3)	
**First time pregnancy, n (%)**			
	No	220 (39.7)	
	Yes	334 (60.3)	

### Compliance Evaluation: Descriptive Compliance Characteristics

**Figure 2:**
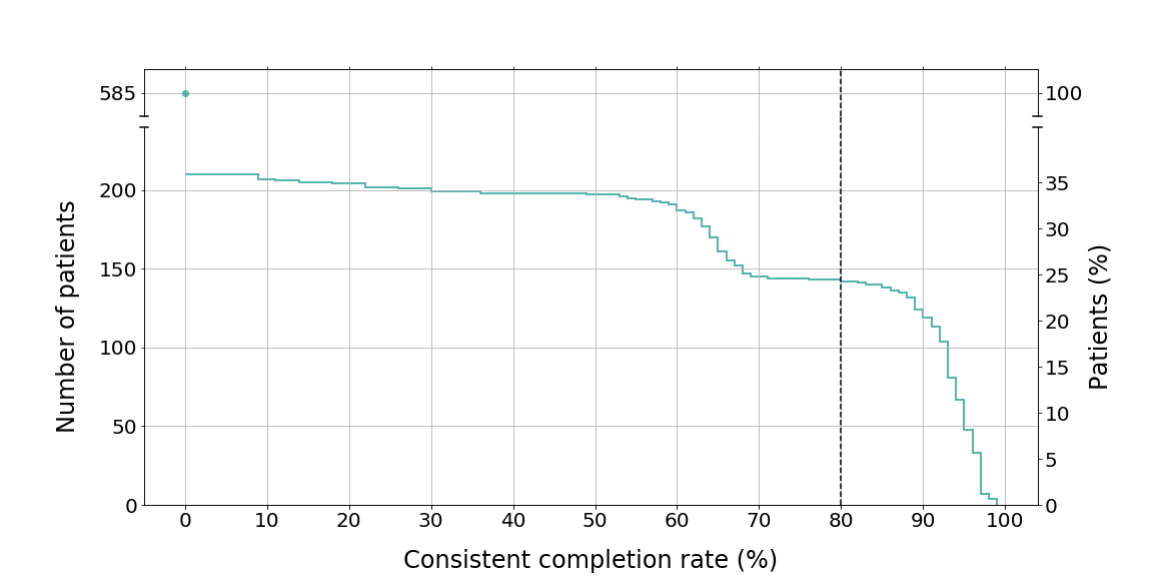
Relationship between completion rate continuity and number of participants.

When applying the definition of a completion rate of ≥80% per visit throughout the study as compliant participation, n=148 patients could be considered as compliant during the prenatal stage of the study, which corresponds to only about 25% of all enrolled participants (Figure 2). Another 63% left at least one visit blank.

As depicted in Table 3, the desired completion rate of 80% to 100% was met by a high number of patients at baseline but decreased significantly in the following assessments. The analysis showed a spike for completion rates of 80% to 100% and 0% to 19%. The range in between these two extremes is evenly distributed and low at every visit analyzed, indicating a tendency to either fill out the entire questionnaire repertoire or not to start the visit altogether.

**Table 3:** Completion rates per visit in relation to the number of participants per visit.

Compliance per visit	Baseline, n (%)	Visit 1, n (%)	Visit 2, n (%)	Visit 3, n (%)	Visit 4, n (%)	Visit 5, n (%)
0%-19%	25 (4.3)	56 (9.6)	216 (36.9)	256 (43.8)	284 (48.5)	321 (54.8)
20%-39%	2 (0.3)	11 (1.9)	6 (1.0)	2 (0.3)	0 (0.0)	8 (1.4)
40%-59%	11 (1.9)	18 (3.1)	11 (1.9)	4 (0.7)	2 (0.3)	8 (1.4)
60%-79%	20 (3.4)	31 (5.3)	17 (2.9)	6 (1.0)	10 (1.7)	40 (6.8)
80%-100%	527 (90.1)	469 (80.2)	335 (57.3)	317 (54.2)	289 (49.4)	208 (35.6)

### Influencing Factors for High Compliance in Online-Based Surveys During Pregnancy

We performed logistic regression analyses on characteristics that could potentially influence compliance (Table 4). Items referring to socioeconomic status appeared to have significant influence; univariate logistic regression analysis showed a positive relationship between higher education (university entrance qualification or higher, *P*=.007), public health insurance (*P*=.01), and origin (other versus German, *P*<.001), as well as a negative correlation with smoking (*P*=.008, current or former smoker versus never smoked). Further medical factors, such as a history of previous abortions or BMI did not show significant correlation with the outcome. Neither computer skills nor the personal review of the user-friendliness of the website had an effect on compliance. Further correlations are depicted in Table 4.

In general, mental health characteristics had no influence on the patients’ compliance. To a small extent, trait anxiety seemed to have an effect, as the STAI-T score at Visit 2 showed a possible negative relationship between trait anxiety and high compliance (*P*=.08). The EPDS score at baseline was not significantly related to the outcome (*P*=.78). Additionally, the intention to deliver via Cesarean section at Visit 1 presented significant negative influence (*P*=.04). The questionnaire characteristics are portrayed in Table 4.

**Table 4:** Univariate logistic regression on high compliance.

Variable		Effect (95% CI)	*P* value
**Socioeconomic characteristics**			
	Age	0.016 (–0.02 to 0.054)	.41
	BMI	0.003 (–0.027 to 0.031)	.84
	Smoker	–0.551 (–0.967 to –0.148)	.008
	Origin	1.333 (0.667 to 2.116)	<.001
	Education	0.521 (0.143 to 0.901)	.007
	Income	0.542 (–0.065 to 1.188)	.09
	Health insurance	0.56 (0.127 to 0.986)	.01
	Employment	–0.189 (–0.75 to 0.34)	.5
	Relationship status	0.262 (–0.154 to 0.695)	.23
**Anamnesis**			
	Previous birth	–0.234 (–0.614 to 0.15)	.23
	Previous miscarriage	0.061 (–0.359 to 0.471)	.77
	Maternal diseases	–0.198 (–0.875 to 0.425)	.55
	Complications	0.034 (–0.346 to 0.41)	.86
	Desire for Cesarian birth	–0.742 (–1.509 to –0.069)	.04
	Sport or birth course	0.199 (–0.221 to 0.565)	.4
**Technical details**			
	Technical skills	0.127 (–0.372 to 0.653)	.63
	User friendly	0.068 (–0.05 to 0.19)	.26
	Medium	–0.187 (–0.499 to 0.12)	.24
**Patient report outcomes**			
	Edinburgh Postnatal Depression Scale result from baseline	–0.007 (–0.055 to 0.04)	.78
	State-Trait Anxiety Inventory (Trait) result from Visit 2	–0.017 (–0.036 to 0.002)	.08
	State-Trait Anxiety Inventory (State) result from Visit 2	–0.019 (–0.041 to 0.004)	.11

Based on the observed correlations, we performed multivariate logistic regression on the factors influencing high compliance in online-based surveys during pregnancy using the variables with significant influence (*P*<.05) from our univariate analysis (Table 4). We determined that German origin (*P*=.006) and smoking (*P*=.02) were the remaining statistically significant variables of our model (Table 5).

**Table 5:** Multivariate logistic regression on high compliance.

Variable	Effect (95% CI)	*P* value
Smoker	–0.538 (–1.001 to –0.088)	.02
Origin	1.099 (0.371 to 1.951)	.006
Education	0.225 (–0.235 to 0.663)	.35
Health insurance	0.248 (–0.268 to 0.755)	.34
Desire for Cesarian birth	–0.597 (–1.381 to 0.1)	.11

### Psychometric Implications of Study Dropout

No significant influence of a change in the EPDS score (*P*=.64) or in the number of physical complaints (*P*=.2) on study termination was found.

## Discussion

### Principal Findings

In this study, we analyzed compliance patterns and determining factors for study compliance among pregnant women in a digital, web-based setting.

Only approximately 25% of patients were highly engaged in the digital app, defined by a completion rate of ≥80% at every visit.

Definitions of compliance vary from number of compliant patients, number of completed visits, to transmission rates; each definition only fits a particular study structure [26,34-36]. We aimed to implement a definition of compliance that included only complete data sets. Therefore, we considered an individual completion rate ≥80% at all visits as compliant. Similar values have been used or reported in other studies, so we considered it to be an appropriate margin [35,37-39].

About 25% of the participants met the aforementioned criteria for high compliance. This compliance rate is comparable to similar studies, which reported compliance rates from 21.8% to 35.6% among pregnant women [40,41]. However, other pregnancy-related studies showed higher compliance ranging from 41% to 92% [22,42,43]. In contrast, at 7%, the number of official dropouts in our study is lower than in comparable studies [16,44,45].

The range between the two extremes of compliance was evenly distributed, indicating patients either accomplished full compliance (80% to 100%) or did not fill out anything (0% to 19%). Technical questions, content-related questions, or difficulties in filling out the online assessment did not seem to play a role in participants’ compliance. However, noncompliant patients may feel overwhelmed with the assessment and may not even start it because of the aforementioned challenges. In this study, we actively encouraged the patients to participate via reminders and asked noncompliant patients over the phone whether they were having problems with the study content or the online portal. In future studies, even more focus must be placed on motivating the patients to simply start the study visit, as they are then more likely to complete the entire visit.

We found a steadily decreasing compliance rather than an abrupt dropout of participants, which could have been provoked by a specific factor that all participants were exposed to at the same time (ie, a shutdown of the website). Other studies have also reported continuously declining compliance rates over time [20,21]. The steadily declining compliance rate can be attributed to a decreasing motivation instead of a low level of initiative or motivation at the beginning of the study, since the number of study participants at baseline was relatively high.

As our key finding, this study revealed that pregnant women who highly engaged with the digital platform had a distinctive profile. We found higher compliance rates in patients with presumed advantageous social standing, which was determined by responses that indicated higher education, higher income, nonsmoking status, private insurance, and being of German origin. Higher socioeconomic advantages such as a certain ethnicity or higher education have previously been reported to be associated with higher compliance [18,20-22]. In Germany, immigrants are still more likely to have a lower socioeconomic status and lower education [46,47]. Hence, our finding reflects a known imbalance for health-related aspects. Before the implementation of a digital risk assessment tool, the needs and expectations of these groups need to be assessed to offer high-quality digital health care to all patients.

Previous and current smoking was also associated with lower compliance. An influence of smoking rate on low compliance has previously been observed in adolescents [48]. Smoking could indicate a limited perception for personal health risks and hence explain a lower interest in health-related studies [49]. A poorer lifestyle and health profile have also been reported to be associated with lower compliance [18].

However, the impact of multicollinearity cannot be completely discounted in socioeconomic analyses. A higher level of education often goes hand in hand with a higher income, a lower likelihood of smoking, and private insurance, which is only available to higher income citizens in Germany [50,51].

Factors influencing compliance in medical research during pregnancy are therefore mostly static in nature and not necessarily pregnancy-specific. Individual solutions may have to be found to improve compliance with digital health solutions.

We also found that a request for a Cesarean section, which was expressed in the second trimester, was associated with lower compliance. Such patients often have misconceptions and a lack of accurate knowledge about different modes of delivery [52,53]. As seen above, our study sample presents with higher levels of education, which may emphasize a lack of accurate information among those with lower education concerning this matter.

Preexisting computer skills or the accessing device (smartphone versus computer) did not show significant influence on user compliance. However, these questions were only implemented at Visit 3, offering limited insight into technical difficulties.

We also evaluated psychometric factors on compliance. The EPDS at baseline and STAI-S at Visit 2 did not have a significant impact on compliance, indicating no detectable correlation between depressive symptoms or state-anxiety. Likewise, Wright et al [21] reported that anxiety, depression, or quality of life did not have an effect on compliance. However, other studies observed higher retention of patients with anxiety or positive affect [22,48], demonstrating the contrasting stance of literature on this topic. Nonetheless, we found a weak link between higher STAI-T scores at Visit 2 and lower compliance (*P*=.08), suggesting a higher burden of the study in patients with preexisting anxious tendencies.

In the multivariate regression model, German origin and being a nonsmoker were revealed to be the influential factors contributing to high compliance. Given the literature and the aforementioned factors on demographic and socioeconomic influence on study compliance [20,48], this result stresses the potential preexisting catering of medical apps to patients of higher socioeconomic standing. As our findings are in accordance with previous research, future studies need to focus on examining the needs of socially underprivileged groups and immigrants in regard to medical studies and online apps. In addition, studies should generally report on the demographic profile of noncompliers as noncompliance can generally cause a bias in results [54]. Customizing educational health apps to patients with lower educational background or offering a service in additional languages may assist in reaching these hard-to-reach groups.

A change in depressive symptoms or an increase in physical complaints had no effect on the abrupt termination of the study, which corresponds to the stance that a poor psychological condition does not promote high compliance [21]. We believe acute dropouts without further notice are a significant problem for studies like this, which was our motivation for an examination of this matter. Therefore, the compliance-determining factors appear to be static characteristics rather than influenceable developments or conditions. We recommend focusing on examining the needs and expectations of participants with a lower socioeconomic background or those of non-German origin to provide all patients with high-quality eHealth solutions.

### Limitations

First of all, with country of origin being the strongest influencing variable, we are faced with the problem of the ambiguous meaning of that variable. We presented this question as “origin” and offered different countries of origin that are common in Germany. We focused on whether or not the participant considered their country of origin to be Germany, which can reflect socioeconomic disadvantages and possibly a lack of pregnancy-related apps in languages other than German [46,47]. In addition to that, the study was only conducted in German, which excluded some demographic groups. However, as a sufficient level of the German language was included in the criteria of eligibility, the influence of a language barrier was minimized. This variable reflects possible disadvantages of immigrants regarding medical studies.

Even though we mainly enrolled women with low-risk pregnancies, the study enrolment took place in a university hospital, which may lead to selection bias of patients with a higher risk for adverse physical or mental health outcomes. Additionally, most of the participants live in the areas surrounding Heidelberg and Tuebingen, which are known to be regions of higher income [55]. This is reinforced by the relatively high number of patients with private health insurance in our study population [56].

Moreover, as with any digital tool, our platform experienced some technical difficulties, mostly due to individual browser settings. These problems were minor and were solved quickly when reported. However, we cannot completely eliminate the possibility of technical difficulties that were not brought to our attention by the participants and may have caused a decline in compliance.

### Conclusions

In conclusion, our results demonstrate that, despite standardized reminders and motivational support, maintaining a high rate of compliance in digital health assessments with pregnant patients over a longer period of time remains challenging. As we observed static characteristics such as socioeconomic background and German origin to be the most influential factors, we propose seeking new ways to reach these groups (e.g., by providing surveys in additional languages and targeting the individuality of every single patient). Further research needs to be directed toward a better understanding of the needs and expectations of socially disadvantaged groups with regard to digital apps.

## Abbreviations

eHealth: electronic health
EPDS: Edinburgh Postnatal Depression Scale
PiiA: “Patient-informiert-interaktiv-Arzt” (patient-informs-interactive-physician)
PRO: patient-reported outcome
SEQ: socioeconomic questionnaire
STAI: State-Trait Anxiety Inventory
